# Items

**Published:** 1879-10

**Authors:** 


### Items.

SCROFULA AND SCARCITY OF FOOD IN LONDON.—We copy the following from one of Dr. L. P. Yandell's letters from London in the *Louisville Medical News:*

*The Children's Hospitals.*—Each of the London hospitals has a separate ward and a daily dispensary for these little people, but besides this, there are more than half a dozen hospitals exclusively for sick children. I have visited the chief hospitals and dispensaries where these unfortunates are brought, and have been afforded every facility by the attending and house physicians for seeing and studying the abundant material of this kind furnished by London's innumerable poor. The in-patients are chiefly surgical cases, and scrofula forms the chief source of their diseases. Of course there are catarrhal, diphtheritic, rheumatic, syphilitic, and malarial cases, and not a few due to inanition, but most of the in-patients are there for scrofula in one form and another. In the out-patients, as in America, the chief source of the acute cases is malaria and the chief source of the chronic cases is scrofula. In the out-patients there are many cases of syphilis, but I see far less infantile syphilis in London than I have seen in France and Germany. Scrofula is the scourge of England to-day. Once the plague was terrible here, and leprosy carried of thousands of English people; but drainage and increased and improved food have banished these diseases. Malarious affections, which, in times past, were as rife in Great Britain as they are now in the unhealthiest parts of America, are much diminished in abundance, and especially are lessened in virulence by the careful surface and under-drainage that have been almost universally carried out. Smallpox, which, a hundred years ago attacked everybody, is now seldom seen. Scurvy, which carried off its thousands of victims annually till Capt. Cook discovered the anti-scorbutic property of fresh vegetables, is now almost unknown; but scrofula was probably never more widespread than at present. Its source, or at least one of its chief sources, is patent to the most careless observer. The English do not get enough fat, enough oleaginous food. This is partly due to the scarcity and costliness of butter, etc., but it is largely due to the popular prejudice against "greasy food," and I am sorry to say this false prejudice is largely shared by the medical men. The profession and the people of the British Isles have yet to learn that fat, oil in some form, or fat-making materials, are just as necessary to human health as oil is to machinery. But, sad to say, not only do the people not get enough fats, but thousands upon thousands get almost no meat, and not a few are even without bread enough to keep down the gnawings of hunger. The other day, an old laborer, seventy-six years old, who had been without work for two weeks, and almost famished for food, cut his throat with a razor. He was discovered dying, and in reply to the inquiry why he did the deed, he said, "Because I can get no work, and I have no home, no food, no friends, nowhere to go." The sapient coroner's jury brought in a verdict of "suicide while temporarily insane." Not only the aged, but the people of middle life and the young lack and suffer hunger in London; and hunger breeds disease.

By the way, have you noticed in the journals the statement that alcohol, malaria and starvation all lead to fatty degeneration, as shown by post mortem investigation? Last year, in one of the English dependencies in India, half the population of natives starved to death, and the English surgeons had a splendid field for post mortem studies.

One word more about oleaginous food. At the very best English dinners, not only is there no butter upon the table, but the vegetables are cooked in plain water, and not a drop of drawn butter is served upon them. Except a bit of fat that you may get with your meat and the oil on the salad, your food is clean of all grease. This is very bad, and Sir Henry Thompson, in two essays upon dining, published in the *Nineteenth Century*, mildly calls attention to the evil.

